# Epac Function and cAMP Scaffolds in the Heart and Lung

**DOI:** 10.3390/jcdd5010009

**Published:** 2018-02-03

**Authors:** Marion Laudette, Haoxiao Zuo, Frank Lezoualc’h, Martina Schmidt

**Affiliations:** 1 Inserm UMR-1048, Institut des Maladies Métaboliques et Cardiovasculaires, Université Toulouse III, 31432 Toulouse, France; marion.laudette@inserm.fr (M.L.); Frank.Lezoualch@inserm.fr (F.L.); 2 Department of Molecular Pharmacology, University of Groningen, 9713AV Groningen, The Netherlands; m.schmidt@rug.nl; 3 Groningen Research Institute for Asthma and COPD (GRIAC), University Medical Center Groningen, University of Groningen, 9713AV Groningen, The Netherlands

**Keywords:** cAMP, Epac, compartmentalization, A-kinase anchoring proteins, phosphodiesterases

## Abstract

Evidence collected over the last ten years indicates that Epac and cAMP scaffold proteins play a critical role in integrating and transducing multiple signaling pathways at the basis of cardiac and lung physiopathology. Some of the deleterious effects of Epac, such as cardiomyocyte hypertrophy and arrhythmia, initially described in vitro, have been confirmed in genetically modified mice for Epac1 and Epac2. Similar recent findings have been collected in the lung. The following sections will describe how Epac and cAMP signalosomes in different subcellular compartments may contribute to cardiac and lung diseases.

## 1. Introduction

In the current manuscript, we aim to highlight the most recent insights into signaling by one of the most ancient second messengers cyclic AMP (cAMP). We focus on novel aspects of cAMP scaffolds maintained by a diverse subset of proteins, among them receptors, exchange proteins, phosphodiesterases, and A-kinase anchoring proteins. We will start with the cardiac system and will then proceed with the lung.

## 2. Epac in Cardiac Disease

Cyclic AMP (cAMP) is one the most important second messengers in the heart because it regulates many physiological processes, such as cardiac contractility and relaxation. The β-adrenergic receptor (β-AR) belongs to the G protein-coupled receptor (GPCR) superfamily, and is essential for the adaptation of cardiac performance to physiological needs. Upon stimulation of β-AR by noradrenaline (released from cardiac sympathetic nervous endings) and circulating adrenaline, cAMP is produced and activates protein kinase A (PKA), which phosphorylates many of the components involved in the excitation-coupling mechanisms, such as L-type the calcium channel (LTCC), phospholamban (PLB), cardiac myosin binding protein C (cMyBPC), and the ryanodine receptor 2 (RyR2), to modulate their activity [1]. Activation of LTCCs produces an inward Ca^2+^ current (ICa) that activates RyR2 through the mechanism known as Ca^2+^-induced Ca^2+^ release (CICR), which raises cytosolic Ca^2+^ concentration and activates contraction. Whereas PKA-dependent LTCC and RyR2 phosphorylation results in mobilization of Ca^2+^ available for contraction, PKA-mediated phosphorylation of phospholamban, a peptide inhibitor of sarcoplasmic reticulum (SR) Ca^2+^-ATPase promotes increased Ca^2+^ reuptake in the SR, thereby removing Ca^2+^ from the cytoplasm and accounting for relaxation [1]. In addition, binding of cAMP to hyperpolarization-activated cyclic nucleotide-gated (HCN) channels that carry the pacemaker current, increases heart rate in response to a sympathetic stimulation (chronotropic effect). From the three β-adrenergic subtypes expressed in the mammalian heart, regulation of cardiac function is ascribed to the β_1_- and β_2_-adrenergic receptor subtypes [2].

Although acute stimulation of the β-AR pathway has beneficial effects on heart function, a sustained activation of β-AR contributes to the development of pathological cardiac remodeling by inducing ventricular hypertrophy, fibrosis, and ultimately, arrhythmia and heart failure (HF), one of the most prevalent causes of mortality globally [3,4,5]. Toxic effects of sustained β-AR stimulation are consistent with the finding that in HF patients, elevated plasma catecholamine levels correlate with the degree of ventricular dysfunction and mortality [6]. However, β-blocker therapy in HF may appear counterintuitive, as catecholamines represent the main trigger of cardiac contractility and relaxation. Indeed, β-blockers restore the adrenergic signaling system which is desensitized by high and chronic concentrations of catecholamines [3]. Thus, it is not so much to block the whole adrenergic signaling which seems important, but rather to modulate its different aspects. It is in this context that several research groups are interested in understanding the role of exchange proteins directly activated by cAMP (Epac) proteins in the development of cardiac arrhythmia and HF [4].

Evidence collected over the last ten years indicates that Epac proteins play a critical role in integrating and transducing multiple signaling pathways at the basis of cardiac physiopathology. Some of the deleterious effects of Epac, such as cardiomyocyte hypertrophy and arrhythmia, initially described in vitro, have been confirmed in genetically modified mice for Epac1 and Epac2. The following sections will describe how Epac signalosomes in different subcellular compartments of the cardiomyocyte may contribute to cardiac disease.

### 2.1. Epac Signalosome in Pathological Cardiac Remodeling

Given the importance of the β-AR-cAMP pathway in cardiac pathophysiology, several studies aim to investigate the role of Epac proteins in the development of cardiac remodeling and HF. Remodeling pathological disorder comprises multiple attacks of which the best described are the modification of the geometry of the cardiac cavity associated with cardiomyocyte hypertrophy, fibrosis, and alterations of calcium handling and energy metabolism [7]. In the long term, these changes affect cardiac contractility and favor progression of HF, a process predominantly relying on cardiac signaling in response to the β_1_-AR subtype.

Among the two Epac isoforms, Epac1 expression was found to be upregulated in various models of cardiac hypertrophy, such as chronic catecholamine infusion and pressure overload induced by thoracic aortic constriction, as well as in the end stages of human HF [8,9]. On the contrary, the anti-hypertrophic action of some hormones and microRNA, including the growth hormone-releasing hormone and microRNA-133, involves Epac1 inhibition [10,11]. A more direct evidence of Epac1’s role in the regulation of cardiac remodeling came from the observation that Epac1 overexpression, or its direct activation with the Epac1 preferential agonist, 8-pCPT-2-*O*-Me-cAMP (8-CPT), increased various markers of cardiomyocyte hypertrophy, such as protein synthesis and hypertrophic genes in primary ventricular myocytes [8,12,13]. It is hypothesized that in the setting of cardiac remodeling, adaptive autophagy antagonizes Epac1-induced cardiac hypertrophy [14]. In vitro studies revealed that the pharmacological inhibition of Epac1 by a tetrahydroquinoline analogue, CE3F4, prevented the induction of cardiomyocyte hypertrophy markers in response to a prolonged β-AR stimulation in rat ventricular myocytes [14,15,16]. These findings indicate that Epac1 signaling may provide a novel means for the treatment of pathological cardiac hypertrophy. It is worth mentioning that Epac1 has also been recently identified as a potential mediator of radiation-induced cardiomyocyte hypertrophy, suggesting that this cAMP-sensor is involved in the side effects of anticancer therapy [17].

**Figure 1:**
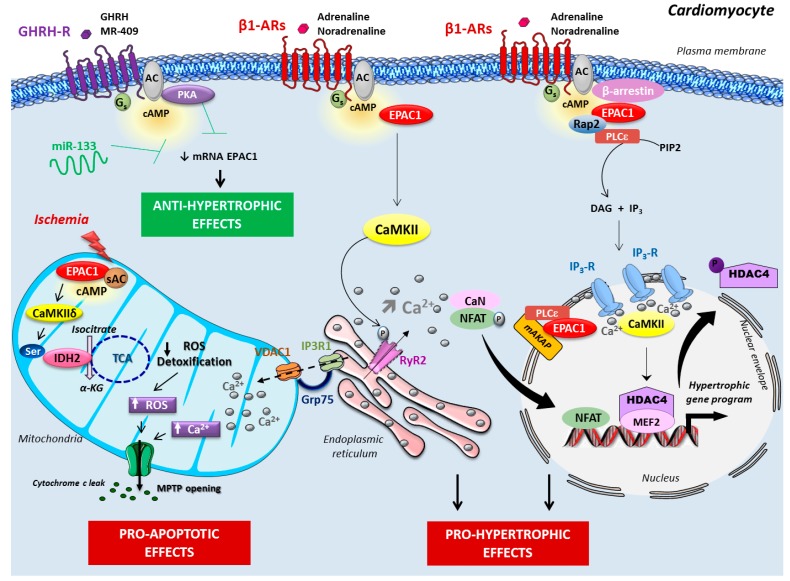
**Epac signalosome in cardiac hypertrophy and ischemia.** Under adrenergic stimulation, the Epac1–β-arrestin complex is recruited at the β_1_-AR, and activates a pro-hypertrophic signaling pathway. Epac1 is also scaffolded at the nuclear envelope with phospholipase C (PLC)ε and muscle-specific A-kinase anchoring proteins (mAKAP) to regulate the hypertrophic gene program. In the nuclear/perinuclear region, PLCε increases nuclear Ca^2+^ content via the activation of the perinuclear IP3 receptor (IP3-R). Epac1 hypertrophic signaling also involves CaMKII-dependent phosphorylation of RyR2, leading to Ca^2+^ leak from the sarcoplasmic reticulum and subsequent calcineurin (CaN) activation. The anti-hypertrophic action of the growth hormone-releasing hormone (GHRH) or its agonistic analog, MR-409, involves the protein kinase A (PKA)-dependent inhibition of Epac1 expression. MicroRNA-133 (miR-133) is cardioprotective, and targets several components of β_1_-AR signaling. In the context of cardiac ischemia, mitochondrial Epac1 (MitEpac1) is activated by cAMP produced by the soluble adenylyl cyclase (sAC), and increases Ca^2+^ overload and ROS accumulation to promote mitochondrial permeability transition pore (MPTP) opening and cardiomyocyte apoptosis. α-KG,α-ketoglutarate; β_1_-AR, β_1_-adrenergic receptor; AC, transmembrane adenylyl cyclase; CaMKIIδ, Ca^2+^/calmodulin-dependent protein kinase II δ-isoform; cAMP, cyclic adenosine monophosphate; DAG, diacylglycerol; GHRH-R, GHRH receptor; GRP75, chaperone glucose-regulated protein 75; HDAC4, histone deacetylase 4; IDH2, isocitrate dehydrogenase 2; IP3, inositol-1,4,5-trisphosphate; IP3R1, IP3 receptor 1; IP3R1, inositol-1,4,5-triphosphate receptor 1; MEF2, myocyte enhancer factor-2; NFAT, nuclear factor of activated T-cells; PIP2, phosphatidylinositol 4,5-biphosphate; ROS, reactive oxygen species; Ser, serine; TCA, tricarboxylic acid cycle; VDAC1, voltage-dependent anion channel 1.

Compelling evidence indicates that Epac1 signalosome is highly compartmentalized and occurs in several micro subcellular compartments, such as the plasma membrane, sarcoplasm, and the nuclear/perinuclear region of cardiomyocytes [8,18,19,20]. A macromolecular complex containing the scaffolding protein β-arrestin, Epac1, and Ca^2+^/calmodulin-dependent protein kinase II (CaMKII), has been reported in the heart [21]. Epac1 constitutively interacts with the β-arrestin in the cytoplasm under basal conditions. Stimulation of β_1_-AR, but not β_2_-AR, induces the recruitment of β-arrestin–Epac1 signaling complex at the plasma membrane, whereby it activates a pro-hypertrophic signaling cascade involving the small GTPases Rap2 and Ras, and CaMKII [22]. This acts as a trigger for histone deacetylase type 4 (HDAC4) nuclear export, which initiates a pro-hypertrophic gene program (Figure 1). Interestingly, Epac1 is prevented from undertaking similar signaling at the β_2_-AR, as the cAMP-hydrolyzing enzyme, phosphodiesterase (PDE)4D5, impedes the interaction of Epac1 with β-arrestin, and therefore its recruitment to activated β_2_-AR. Of particular importance, disruption of PDE4D5–β-arrestin complex formation with a cell-permeant peptide promotes binding of Epac1–β-arrestin to β_2_-AR and, consequently β_2_-AR signaling switches to a β_1_-AR-like pro-hypertrophic signaling to increase cardiac myocyte remodeling [22]. Taken together, these data provide evidence that Epac compartmentalization contributes to the functional differences between cardiac β-AR subtypes.

Besides its sarcolemma distribution, Epac1 is also concentrated in the nuclear/perinuclear region of cardiomyocytes, positioned well to regulate nuclear signaling [8,20]. Specifically, it was shown that Epac1 is scaffolded at the nuclear envelope with phospholipase C (PLC)ε and muscle-specific A-kinase anchoring proteins (AKAPs) to regulate the hypertrophic gene program in primary cardiomyocytes [19,23,24] (Figure 1). Interestingly, a detailed analysis of Ca^2+^ mobilization in different microdomains demonstrated that Epac (probably Epac1) preferentially elevated Ca^2+^ in the nucleoplasm, correlating with the perinuclear/nuclear localization of Epac1 [25]. Additional in vitro studies showed that Epac1, via its downstream effector, the small G protein Rap2, activated PLC to promote the production of inositol 1,4,5-trisphosphate (IP_3_) [26]. Based on this finding, a working hypothesis has been proposed, whereby Epac1 can activate PLC, causing nuclear Ca^2+^ increase via perinuclear IP_3_ receptor (IP3-R), which results in the activation of Ca^2+^-dependent transcription factors involved in cardiac remodeling [25,27] (Figure 1). Consistently, in cultured cardiomyocytes, it has been reported that Epac activates CaMKII to induce the nuclear export of HDAC4 de-repressing the transcription factor myocyte enhancer factor 2 (MEF2) which activates gene transcription, essential for the hypertrophic program [25,26]. Collectively, these findings point to Epac1 role in activating the excitation–transcription coupling, the process by which Ca^2+^ activates gene transcription [27]. Additional Epac hypertrophic signaling have been described and include the GTPase H-Ras, the Ca^2+^ sensitive protein, calcineurin, and its downstream effector, nuclear factor of activated T cells (NFAT), which are key mediators of cardiac remodeling [8,26].

More recently, the study of Epac gene deleted mice has made it possible to better understand the role of these proteins in cardiac pathological remodeling. Global knockout (KO) mice for Epac1 or Epac2, or double full KO for Epac1 and Epac2, do not present any cardiac abnormality, suggesting that these guanine-nucleotide exchange factors activated by cyclic adenosine monophosphate (cAMP-GEFs) do not play a major role during cardiac development [14,28,29]. None of the deletions appreciably affected basal cardiac function. Although Epac has been shown to influence myofilament Ca^2+^ sensitivity in rat cardiomyocytes [18,30], the effects of Epac activation in cell contractility remain controversial, and may depend on the steady-state Ca^2+^ levels at which the myocyte is functioning [18,31,32,33]. Overall, Epac proteins do not play a major role in the physiological regulation of cardiomyocyte contractility in response to acute β-AR stimulation, compared with PKA, which is the main cAMP effector in this process [29,34]. However, Epac1 genetic inhibition specifically reduces cardiac remodeling induced by chronic activation of β-AR, which confirms the importance of Epac1 in the β-adrenergic signaling during cardiac stress condition [14,28]. Moreover, Epac1 deleted cardiomyocytes prevented 8-CPT-dependent HDAC5 translocation, consistent with its involvement in pathological hypertrophy [20]. Of note, in another model of cardiac hypertrophy induced by aortic stenosis, Epac1 knockdown fails to prevent cardiac hypertrophy, but only fibrosis and cardiomyocyte apoptosis, suggesting that the cardioprotective effects of Epac1 deletion with respect to hypertrophy depend on the nature of stress [19].

### 2.2. Role of Epac in Heart Failure and Arrhythmia

Interestingly, Epac1 KO mice show better cardiac contractility (maintenance of the inotropic reserve) and decreased susceptibility to HF in response to different hypertrophic stress conditions (catecholamine infusion or myocardial pressure overload) [14,28]. Further evidence for the cardioprotective effect of Epac1 inhibition came from the recent report that Epac1 deficiency attenuates type 5 adenylyl cyclase-mediated catecholamine stress-induced cardiac dysfunction [35]. It is interesting to note that Epac1 and Epac2 deleted mice are protected from the incidence of atrial and ventricular arrhythmia, respectively, suggesting a specific role of Epac isoforms in cardiac rhythm disorders [28,29]. Conversely, direct pharmacological activation of Epac with the cAMP analogue 8-CPT promotes ventricular arrhythmogenesis in isolated perfused mouse hearts [36]. Such arrhythmogenic features were also observed in rat cardiomyocytes, but after sustained Epac activation [37]. Yet, Brette and colleagues reported that 8-CPT induced an action potential lengthening in rat ventricular myocytes, a process involved in the genesis of arrhythmia by predisposing cardiac myocytes to early after depolarizations and dispersion of repolarization [38].

**Figure 2:**
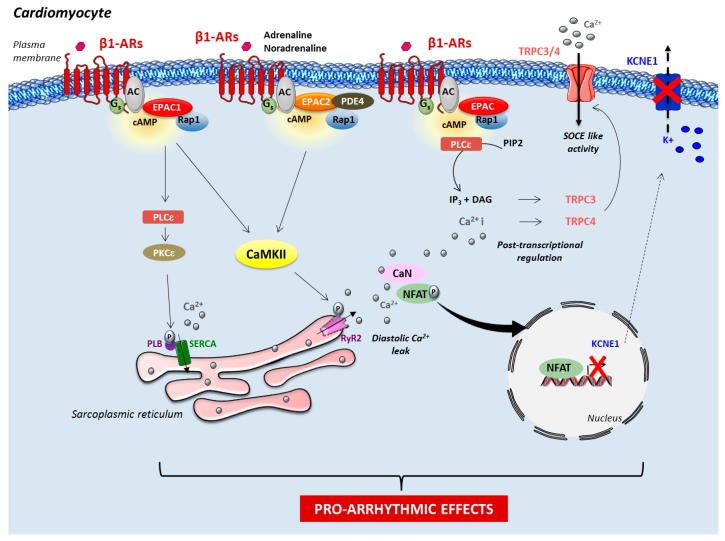
**Role of Epac in cardiac arrhythmia.** Epac proteins increase the phosphorylation state of the ryanodine receptor 2 (RyR2) via CaMKII, and subsequent Ca^2+^ leak from the sarcoplasmic reticulum (SR) may trigger arrhythmia. Epac1-induced hyperphosphorylation of phospholamban (PLB) may also contribute to the development of arrhythmia and heart failure. In addition, Epac enhances store-operated Ca^2+^ entry (SOCE)-like activity, which is related to an increased amount of functional transient receptor potential canonical (TRPC) 3 (TRCP3) and TRCP4 channels. This additional Ca^2+^ entry pathway in the cardiomyocyte and the downregulation of the potassium voltage-gated channel subfamily E member 1 (KCNE1) participate in the proarrhythmic effect of Epac proteins. β_1_-AR, β_1_-adrenergic receptor; AC, transmembrane adenylyl cyclase; CaMKII, Ca^2+^/calmodulin-dependent protein kinase II; cAMP, cyclic adenosine monophosphate; CaN, Calcineurin; DAG, diacylglycerol; IP3, inositol-1,4,5-trisphosphate; P, phosphorylation; PDE4, phosphodiesterase 4; PIP2, phosphatidylinositol 4,5-biphosphate; PKCε, protein kinase C epsilon type; PLB, phospholamban; PLCε, phospholipase C epsilon type; SERCA, sarcoendoplasmic reticulum calcium transport ATPase.

Mechanistically, few studies demonstrated that mainly Epac1 regulated the expression level of proarrhythmic channels, such as the slow delayed-rectifier potassium K+-current (IKs) subunit potassium voltage-gated channel and transient receptor potential canonical 3 and 4 channels that enhance store-operated Ca^2+^ entry [39,40] (Figure 2). Epac function may also affect susceptibility to arrhythmia via the regulation of gap junction formation [41,42]. Importantly, in isolated ventricular myocytes, activation of Epac with either 8-CPT or β_1_-AR induces a spontaneous release of Ca^2+^ from the SR (a process named Ca^2+^ sparks) via the CaMKII-dependent phosphorylation of RyR2 on Serine 2814 or 2815 (depending on the species), thereby causing diastolic Ca^2+^ leak in a PKA-independent manner [29,31,32,33,43] (Figure 2). Consistent with the localization of Epac2 along T tubules in mouse cardiomyocytes [20], it has been proposed that the increase of ectopic release of Ca^2+^ following Epac2 (and not Epac1) activation by β_1_-AR, could be the cause of arrhythmogenic effects in cardiomyocytes [29]. The recent finding that SR Ca^2+^ leak observed upon PDE4 inhibition involves Epac2 suggests that the interaction of PDE4 and Epac2 are critical for coordinating the pro-arrhythmic effect of cAMP [34]. Adding complexity to the matter, another study showed that Epac1 promoted PLB hyperphosphorylation on Serine 16 via PKCε [28]. This could lead to SR Ca^2+^ overload with Ca^2+^ leak and subsequent arrhythmia [28] (Figure 2). Based on the aforementioned studies, the beneficial effect of Epac inhibition seems, therefore, very attractive for the development of novel therapies against HF and arrhythmia. However, few controversies have been reported in the literature. Among them, Yang and colleagues recently reported that pharmacological inhibition of Epac2 with ESI-05 was proarrhythmic in rat [44]. Further pharmacological and genetic studies combining the use of Epac isoform-specific ligands and conditional Epac KO mice are required to better decipher the role of Epac isoforms in cardiac rhythm disorders.

### 2.3. Role of Mitochondrial Epac in Cardiac Ischemia

Acute myocardial infarction is a leading cause of mortality and morbidity worldwide. Early coronary reperfusion has been established as the best therapeutic strategy to limit infarct size and improve prognosis. However, the process of reperfusion can itself induce cardiomyocyte death, known as myocardial reperfusion injury (I/R), for which there is still no effective therapy [45,46]. Mitochondria have been recognized as playing a central role in both apoptotic and necrotic cell death [47]. Indeed, during I/R injury, cardiomyocyte death is initiated by mitochondrial Ca^2+^ overload and an excessive production of reactive oxygen species (ROS) which trigger the mitochondrial permeability transition pore (MPTP) opening, resulting in mitochondrial depolarization, swelling, and rupture of the external mitochondrial membrane. This leads to the uncoupling of the respiratory chain, and the efflux of cytochrome c and other proapoptotic factors that may induce apoptosis or necrosis [48].

Depending on the nature of the stimulus and the cell type used in the study, Epac may play a proapoptotic or antiapoptotic role [49]. For instance, in neonatal rat cardiomyocytes, Epac cooperates with PKA in the antiapoptotic effects of exendin-4, a glucagon-like peptide-1 receptor agonist [50]. Similarly, activation of both PKA and Epac with cAMP analogues confers cardioprotection against I/R injury in isolated rat heart [51]. Interestingly, it is suggested that long-term feeding of an obesogenic high fat diet renders the myocardium less susceptible to I/R induced injury via Epac-dependent signaling [52]. Yet, recent findings using isolated cardiomyocytes from ischemic rat hearts have implied that the cardioprotective effect induced by urocortin-1 involved the Epac2 pathway [53]. On the contrary, in vivo experiments showed that Epac1 genetic ablation in mice protected against myocardial I/R injury with reduced infarct size and cardiomyocyte apoptosis [54]. Consistent with an earlier finding showing the mitochondrial expression of transfected Epac1 in COS-7 cells [55], Epac1 is expressed in the mitochondrial inner membrane and matrix of cardiomyocytes. A form of Epac1 deleted in its mitochondrial-targeting sequence protects against hypoxia/reoxygenation (a condition mimicking in vivo I/R)-induced cell death, indicating that mitochondrial Epac1 participates in cardiomyocyte death during hypoxic stress [54]. Mechanistic studies demonstrated that during hypoxia/reoxygenation, Epac1 was activated by the type 10 soluble adenylyl cyclase (sAC) to increase mitochondrial Ca^2+^ uptake and ROS production, thereby promoting mitochondrial death signaling, such as MPTP opening, cytochrome c release, and both caspase-9 and -3 activation [54]. However, these results are not in agreement with another study, which reported that direct activation of sAC with HCO_3_^−^ prevented Ca^2+^-induced MPTP opening through Epac1, suggesting that Epac1 might protect from cardiomyocyte death [56]. The higher amount of cAMP produced in the model of hypoxia/reoxygenation, and subsequent massive increase in mitochondrial ROS and Ca^2+^ levels, could potentially account for the observed differences.

Interestingly, we found that Epac1 is highly compartmentalized in mitochondria and targets key proteins involved in mitochondrial Ca^2+^ uptake and ROS production. Indeed, firstly, we revealed that Epac1 interrelated with a macromolecular complex composed of the VDAC1 (voltage-dependent anion channel 1), the GRP75 (chaperone glucose-regulated protein 75), and the IP3R1 (inositol-1,4,5-triphosphate receptor 1). This complex localized at the endoplasmic reticulum (ER) junction is considered as a hot spot from Ca^2+^ transfer from the ER to the mitochondria [57]. Under hypoxic condition, Epac1 activation increased the interaction with the VDAC1/GRP75/IP3R1 complex, hence facilitating ER to mitochondrial Ca^2+^ transfer. Epac1-mediated mitochondrial Ca^2+^ overload subsequently provoked MPTP opening, cytochrome c release, and ultimately, cardiomyocyte death [54] (Figure 1). Secondly, our study revealed a key role for Epac1 in the accumulation of mitochondrial ROS production during hypoxia. Surprisingly, we observed that Epac1 imported CaMKII into matrix where they formed a multi-molecular complex with isocitrate dehydrogenase 2 (IDH2), a critical mitochondrial enzyme of the tricarboxylic acid (TCA) cycle involved in ROS detoxification [54]. Mitochondrial Epac1 negatively regulates via the CaMKIIδ-dependent phosphorylation activity of IDH2, and hence, decreases the antioxidant capabilities of the cardiomyocytes during I/R [54] (Figure 1). Altogether, these findings identify Epac1 as a central mechanism for mitochondrial Ca^2+^ entry and ROS production in myocardial cell death, and indicate that mitochondrial-targeted Epac1 inhibition could prevent or reduce myocardial death in the setting of cardiac ischemia.

## 3. Chronic Obstructive Pulmonary Disease 

Chronic obstructive pulmonary disease (COPD) is one major health problem known to increase morbidity and mortality all over the world. It is predicted that COPD will become the third leading cause of death (~ 8.3 million), and the third leading cause of death by disability until 2030 [58]. Globally, exposure to cigarette smoke (CS) is considered to be the primary cause for COPD. Inhalation of CS causes the release of different cytokines, chemokines, and lipid mediators (such as tumor necrosis factor-α (TNF-α), interleukin 8 (IL-8), transforming growth factor-β (TGF-β) and leukotriene B4) from resident cells in the lung including epithelial cells and alveolar macrophages. Subsequently, these mediators activate inflammatory cells which release large amounts of proteases, including elastase and matrix metalloproteinases (MMPs), into the matrix compartment, thereby triggering the complex process of remodeling, thus leading to obstruction of small airways, emphysema, with enlargement of air spaces and destruction of lung parenchyma, loss of lung elasticity, and closure of small airways, fibrosis, inflammation, mucus hyper-secretion, and pulmonary hypertension. Furthermore, more and more evidence indicates that CS exposure also provokes an oxidant/antioxidant imbalance, which in turn will subsequently induce COPD exacerbations [59,60]. Therefore, the most effective way to prevent the development of COPD is smoke cessation [61,62]. Additionally, other factors including exposure to indoor air pollutions from biomass fuels, particularly in developing countries, occupational dusts, chemicals, and genetics, may also contribute toward disease morbidity and mortality [63,64]. Currently, the pharmacological management of COPD mainly relies on bronchodilator therapy, mainly β_2_-agonists and anticholinergics, by activating different signaling pathways [65,66,67,68]. β_2_-Agonists induce airway smooth muscle (ASM) cell relaxation through enhanced intracellular cAMP production, whereas anticholinergics or antimuscarinic drugs antagonize muscarinic receptors (M1, M2 and M3) to a certain extent, thus inhibiting ASM contraction, due to the reduction of intracellular Ca^2+^. PDE4 inhibitors, which mediates cAMP breakdown (see below), are also approved to be used as an add-on treatment for severe COPD patients associated with bronchitis and a history of frequent exacerbations [69]. In addition, anti-inflammatory drugs, such as inhaled glucocorticosteroids, are often used, mainly in patients with frequent exacerbations [70].

### 3.1. Compartmentalization of cAMP in the Lung

**Figure 3:**
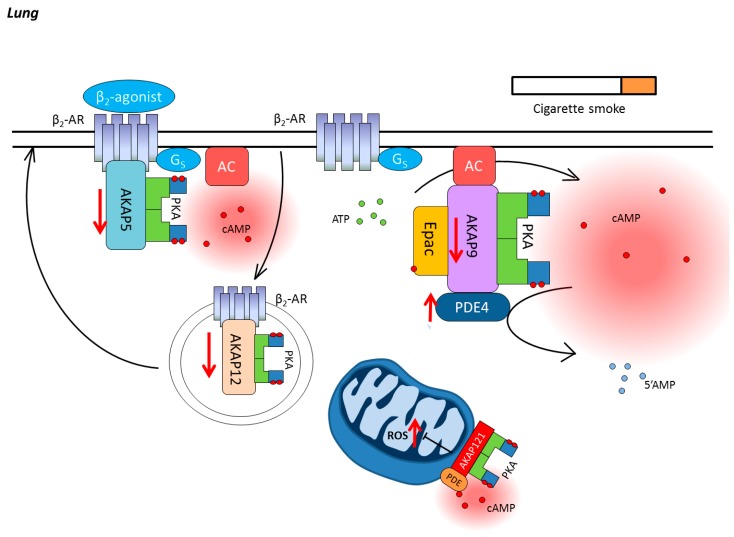
**cAMP compartmentalization in COPD.** As one of main inducing factors, cigarette smoke (CS) is able to modulate numerous molecular signals in both structural and inflammatory cells in the lung. CS decreases the expressions of A-kinase anchoring protein (AKAP)5 and AKAP12, thus, regulating the effect of β_2_-agonists on COPD pathological development. Moreover, CS interferes with cAMP compartments by AKAP9, which binds and regulates the function of adenylyl cyclases (ACs). In addition, intracellular cAMP concentration is further decreased by upregulating cAMP hydrolyzing enzyme PDEs expression. β_2_-AR, β_2_-adrenergic receptor; AC, transmembrane adenylyl cyclase; cAMP, cyclic adenosine monophosphate; ATP, adenosine triphosphate; PDE4, phosphodiesterase 4; AKAP, A-kinase anchoring protein; PKA, protein kinase A; Epac, exchange protein directly activated by cAMP; ROS, reactive oxygen species.

The production of cAMP is initiated by the stimulation of Gs-coupled receptors, such as the β-AR and distinct subset of prostanoid receptors [71]. After receptor ligand binding, ACs are activated by the α subunit of the Gs-protein, thus resulting in cAMP synthesis from adenosine triphosphate (ATP) [72]. Intracellular cAMP levels are tightly controlled by cyclic nucleotide PDEs, which hydrolyze cAMP to 5′-AMP, and thereby terminate its signaling [73]. Membrane clustering of Gs-coupled receptors ACs and PDEs, which are localized in lipid rafts and caveolae, together with cAMP downstream effectors, such as cAMP-gated ion channels, PKA, and Epac, dynamically regulate intracellular cAMP signaling in the lung, including airway relaxation [74,75], reduction of inflammation [76,77,78,79], and fibrosis [77,80]. In addition, AKAPs bind directly to PKA and its targeted proteins, and physically tether these multi-protein complexes to specific locations, generating spatiotemporal discrete signaling complexes [81,82], and subsequently controlling specific cellular responses (Figure 3).

### 3.2. Cyclic Nucleotide Phosphodiesterases in COPD

PDEs, which comprise 11 family members and at least 21 isoforms with different splice variants [83], are able to hydrolyze cyclic nucleotides (cAMP and cGMP) to their inactivate 5′ monophosphates within subcellular microdomains, thereby modulating cyclic nucleotide signaling pathways.

PDE4 is the most extensively studied PDE, and it is widely expressed in almost all different kinds of cells in the lung. From a clinical viewpoint, there are dramatic differences in the PDE4 isoforms’ expression in inflammatory cells of smokers with COPD, smokers without COPD, and nonsmokers [84]. PDE4A4 was significantly upregulated not only in lung macrophages from COPD patients, but also in peripheral blood monocytes of smokers, together with PDE4B2 [84]. In isolated peripheral blood neutrophils, significantly higher PDE4B and PDE4D, but not PDE4A or PDE4C, mRNA levels could be observed in the COPD patients compared to healthy subjects. Yoon et al. reported the association of a novel PDE4D single nucleotide polymorphism (rs16878037) with COPD from a genome-wide association study [85]. PDE4 is also of importance in other pulmonary diseases, such as asthma. It was shown by Trian et al. that β-agonist isoproterenol-induced cAMP production in asthmatics ASM cells was dramatically decreased due to increased PDE4D expression, rather than an alteration in PDE3A or PDE5A expression [86]. Furthermore, CS, as the primary cause for COPD, was also proven to increase PDE4 isoforms’ expression and activity in different experimental settings. Higher PDE4B and PDE4D mRNA levels could be detected after 6 h CS extract exposure in isolated peripheral blood neutrophils [87]. Exposure with CS extract for 24 h upregulated PDE4 activity in differentiated bronchial epithelial cells, with a markedly increased mRNA transcripts for PDE4B, while increments in PDE4A and D transcripts remained below significance [88].

Other PDE family members also attracted the attention of researchers, such as PDE7, which is encoded by PDE7A and PDE7B. PDE7B is expressed predominantly in brain, heart, and liver, but not in lung [89,90]. However, PDE7A, which is widely expressed in airway structural cells, including airway epithelial cells [91], ASM cells and lung fibroblast, and also human proinflammatory and immune cells [92], has particularly drawn attention in the treatment of COPD [93]. It has been shown that the protein expression of PDE7A1 is significantly increased in human monocytes during cell aging [94]. In addition, PDE3A is upregulated in human endobronchial biopsies obtained from patients with asthma, indicating that PDE3 is also involved in the pathogenesis of lung diseases.

### 3.3. AKAPs in COPD

As one of the most vital pharmaceutical targets in COPD, β_2_-AR directly interacts with AKAP5 and AKAP12, which modulate either the downstream extracellular signal-regulated protein kinase (ERK)1/2 activation or receptor resensitization [81,94,95,96]. Therefore, it is believed that AKAP5 and AKAP12 play a pivotal role in modulating the effect of β_2_-agonists on COPD pathological development. Poppinga et al. showed that less AKAP5 and AKAP12 proteins could be detected in CS-exposed ASM cells, which was also confirmed using lung tissues from COPD patients [97]. Moreover, st-Ht31, which disrupts global AKAP–PKA interactions (see Figure 3), increased the IL-8 secretion induced by CS exposure in human ASM cells, and reduced the suppression of β_2_-agonist fenoterol through disturbed ERK signaling [97]. On the contrary, AKAP5 and AKAP12 expression were not significantly changed by CS in human bronchial epithelial cells [98], indicating a different regulatory pathway involved other AKAP members in epithelial cells (Figure 3). Indeed, it has been demonstrated that AKAP9, which binds and regulates ACs [99,100], was able to maintain epithelial barrier function [99] (Figure 3). In addition, as a major site for energy generation and reactive oxygen species (ROS) production, mitochondria cAMP compartments was regulated by a set of mitochondrial AKAPs. In particular, AKAP121, tethering PKA to the outer wall of mitochondria, play a pivotal role in mitochondrial function maintenance and keeping the tolerance to oxidative stress in vascular smooth muscle cells [101], indicating a potential protective effect on CS-induced ROS production for future studies (Figure 3).

### 3.4. Epac in COPD

In addition to PKA, as mentioned earlier, Epac is another downstream effector for cAMP [102]. Epac, which is able to be activated by PKA or cAMP in both a PKA-dependent and -independent manner, has been proven to associate with diverse effectors, thus contributing to numerous cellular processes, including airway relaxation, cytokine secretion, barrier function, cell proliferation, migration, and protein translocation in the lung. Also, it has been demonstrated by Scott et al. that Epac activation by 8-CPT could reverse neutrophils’ phagocytic impairment induced experimentally by using β_2_-agonists, without interfering with RhoA activity [103]. In addition, Epac activation by 8-CPT changed methacholine-induced myosin light chain (MLC) phosphorylation in ASM cells, skewing the balance between RhoA and Rac1 towards Rac1, and thus reducing the phosphorylation of MLC; a process leading to ASM relaxation [75]. In addition, pharmacological activation of Epac attenuated CS extract-induced IL-8 release from human ASM cells by activating the NF-κB inhibitory protein IκBα and inhibiting p65 nuclear translocation, underling the inhibitory effect of Epac on NF-κB activation [104]. Two isoforms, Epac1 and Epac2, are expressed in most lung cell types, among them, ASM, epithelial cells, fibroblasts, and some immune cells [103,105]. Intriguingly, a selective downregulation of Epac1, rather than Epac2, expression was observed in both CS-exposed ASM cells and lung tissue from COPD patients, pointing to distinct intracellular functions and locations of Epac1 and Epac2 [104]. It was further demonstrated that the upregulation of miRNA7 expression in COPD patients was linked to the downregulation of Epac1 [106]. Moreover, the role of Epac1 and Epac2 was further investigated in Epac1(−/−) and Epac2(−/−) mice using an acute and short-term CS exposure model. Compared to wild type mice exposed to CS, Epac1(−/−) mice showed increased MUC5AC and matrix remodeling parameters (TGF-β, collagen I, and fibronectin) in the lung homogenates, however, Epac2(−/−) mice had lower amount of inflammatory cells (total inflammatory cells, macrophages, and neutrophils) in the bronchoalveolar lavage fluid, suggesting that Epac1 was able to inhibit remodeling process, whereas Epac2 primarily increased inflammatory processes [107]. These differences between Epac1 and Epac2 indicate compartmentalized cAMP signaling in the lung, which needs to be further studied in the future.

## 4. Conclusions and Future Perspectives

Evidence collected over the last decade indicates that Epac and the cAMP signalosome substantially contribute to the development and progression of heart and obstructive lung disorders, as exemplified here by studies focusing on COPD. Traditionally, research focused on PKA as one of the main targets of cAMP. However, recent studies indicated that next to classical signaling pathways, there seem to be substantial role for additional cAMP targets, such as Epac, and for the theme of compartmentalized cAMP signaling. The latter topic involves next to cAMP-elevating receptors (for example the β-ARs), cAMP-producing adenylyl cyclase and cAMP-degrading PDEs, a subset of AKAPs. Particularly, studies on the levels of Epac and PDEs benefit from the recent development of pharmacological tools [13,19,108], suggesting that the therapeutic arsenal to treat chronic disorders of the heart and the lung will be substantially improved in the next decades. Unfortunately, the research on compartments being stabilized and maintained by members of the AKAP superfamily still lacks the development of subtype specific modulators, which will hopefully be the focus of future studies.

In the heart, research over the last years has shown that particularly Epac1 seems to act cardioprotectively with regard to cardiac fibrosis and apoptosis, but that the role of Epac1 seems to be rather distinct, and being largely dependent on the nature of the stress inducer. Such findings nicely reflect the fact that our aging world population requires a vast majority of novel personalized medicine-based drugs [19,71,109]. Distinct roles of Epac1 and Epac2 seemed to emerge in the field of heart failure and arrhythmia, as well as cardiac ischemia. Here, interaction of Epac with members of the AKAP family or PDE subtype, such as PDE4, seem to be leading next to a defined subcellular targeting of Epacs to defined compartments, such as the nucleus and mitochondria. In the lung, it seemed to be that PDEs are crucially involved in the compartmentalization of cAMP next to members of the AKAP family and Epac. Interestingly, it seemed to be that subtypes of PDEs guide cAMP properties in a rather response-specific manner, such as anti-inflammation and bronchodilation. Novel anti-inflammatory drugs are urgently required, as under disease conditions being characterized by an overload of ROS, the golden treatment standard, glucocorticosteroids, are ineffective. Most likely, compartment-specific mode of actions of PDEs are supported by AKAPs and Epac. Future studies should focus on unravelling compartmentalized signaling cues in the heart and lung.
